# Risk Models for Adverse Events in Microsurgery for Intracranial Unruptured Aneurysms

**DOI:** 10.1227/neu.0000000000003867

**Published:** 2025-12-05

**Authors:** Victor E. Staartjes, Alexios A. Adamides, Pasquale Anania, Mustafa K. Baskaya, Vladimir Beneš, Amir R. Dehdashti, Antonio Di Ieva, Paolo Ferroli, Asgeir S. Jakola, Giuseppe Lanzino, Michael T. Lawton, Ondra Petr, Thomas Petutschnigg, Giampietro Pinna, Dino Podlesek, Ivan Radovanovic, Veit Rohde, Nico Stroh-Holly, Carmelo Lucio Sturiale, Anthony C. Wang, Luca Regli, Giuseppe Esposito, for the PRAEMIUM Investigators, Carlo Serra

**Affiliations:** 1 Machine Intelligence in Clinical Neuroscience & Microsurgical Neuroanatomy (MICN) Laboratory, Department of Neurosurgery, Clinical Neuroscience Center, University Hospital Zurich, University of Zurich, Zürich, Switzerland;; 2 Department of Clinical Neuroscience, Karolinska Institutet, Stockholm, Sweden;; 3 Department of Neurosurgery, The Royal Melbourne Hospital, Melbourne, Victoria, Australia;; 4 Department of Neurosurgery, IRCCS Ospedale Policlinico San Martino, Genoa, Italy;; 5 Department of Neurosurgery, University of Wisconsin, Madison, Wisconsin, USA;; 6 Department of Neurosurgery, Central Military Hospital, Charles University, Prague, Czech Republic;; 7 Department of Neurosurgery, North Shore University Hospital, Northwell Health, Manhasset, New York, USA;; 8 Computational NeuroSurgery (CNS) Lab, Macquarie Medical School, Macquarie University, Sydney, Australia;; 9 Department of Neurosurgery, Istituto Neurologico Carlo Besta, Milan, Italy;; 10 Department of Neurosurgery, Sahlgrenska University Hospital, Institute of Neuroscience and Physiology, Sahlgrenska Academy, Gothenburg, Sweden;; 11 Department of Neurosurgery, Mayo Clinic, Rochester, Minnesota, USA;; 12 Department of Neurosurgery, Barrow Neurological Institute, Phoenix, Arizona, USA;; 13 Department of Neurosurgery, University Hospital Innsbruck, Innsbruck, Austria;; 14 Department of Neurosurgery and Stroke Research Center Bern, Inselspital, Bern University Hospital, University of Bern, Bern, Switzerland;; 15 Department of Neurosurgery, University Hospital Verona, Verona, Italy;; 16 Department of Neurosurgery, University Hospital Dresden, Dresden, Germany;; 17 Department of Neurosurgery, Toronto Western Hospital, Toronto, Ontario, Canada;; 18 Department of Neurosurgery, University Hospital Göttingen, Göttingen, Germany;; 19 Department of Neurosurgery, Kepler University Hospital, Linz, Austria;; 20 Fondazione Policlinico Universitario A. Gemelli IRCCS, Università Cattolica del Sacro Cuore, Rome, Italy;; 21 Department of Neurosurgery, University of California Los Angeles, Los Angeles, California, USA

**Keywords:** Predictive analytics, Outcome prediction, Machine learning, Aneurysm, Unruptured intracranial aneurysm, Neurosurgery

## Abstract

**BACKGROUND AND OBJECTIVES:**

Preventive treatment of unruptured intracranial aneurysms (UIAs) requires assessment of treatment risks vs expected benefit. Although established scores exist to estimate rupture and growth risk, currently, no externally validated tools exist to estimate the risks of microsurgical treatment of UIAs. Clinical prediction models based on machine learning enable generation of personalized risk estimates for each individual patient based on their specific patient and aneurysm characteristics.

**METHODS:**

Using data from 20 international centers from the prediction of adverse events after microsurgery for intracranial unruptured aneurysms study on patients treated microsurgically for UIAs, we developed and externally validated clinical prediction models for 3 outcomes measured at hospital discharge: poor neurological outcome (modified Rankin Score ≥3), new sensorimotor neurological deficits, and all-cause adverse events (Clavien-Dindo Grade ≥1).

**RESULTS:**

A total of 3705 patients were included. Data from 13 centers (2881, 78%) were used for model development. Fully trained models were evaluated on 824 patients (22%) from 7 additional centers. Average age was 56 ± 12 years, and 1049 (28%) were male. At discharge, poor neurological outcome was seen in 514 patients (14%). New sensorimotor deficits were observed in 534 patients (14%), and 894 patients (24%) experienced adverse events until discharge. At external validation, prediction of poor neurological outcome was achieved with good calibration and an area under the curve (AUC) = 0.70 (95% CI: 0.63-0.75). Similarly, new neurological deficits were predicted with good calibration and with an AUC = 0.69 (95% CI: 0.63-0.74). Prediction of all-cause adverse events only achieved an AUC = 0.59 (95% CI: 0.55-0.64) with fair calibration. The prediction model was integrated into a web application accessible at https://neurosurgery.shinyapps.io/PRAEMIUM/.

**CONCLUSION:**

The developed models for prediction of poor neurological outcome and new sensorimotor neurological deficits at discharge exhibit good calibration and fair discrimination based on a multinational external validation, indicating that the predicted probabilities correspond well to real-world risks and may thus be clinically useful in more objectively estimating the risk of microsurgical treatment.

## Graphical abstract

**Figure:**
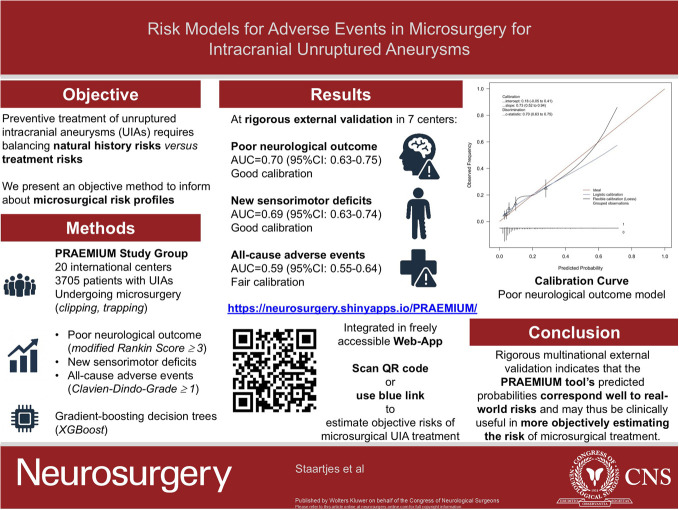

**ABBREVIATIONS:**
CDG: Clavien-Dindo grading
ELAPSS: Earlier subarachnoid hemorrhage, Location of aneurysm, Age, Population, Size of aneurysm, Shape of aneurysm score
PHASES: Population, Hypertension, Age, Size of aneurysm, Earlier subarachnoid hemorrhage, Site of aneurysm score
PRAEMIUM: prediction of adverse events after microsurgery for intracranial unruptured aneurysms
UIATS: Unruptured Intracranial Aneurysm Treatment Score.

Incidental detection of unruptured intracranial aneurysms (UIAs) is increasing markedly because of the rise in availability of cranial imaging.^1^ Consequently, patients and clinicians are frequently faced with complex decisions regarding treatment vs observation in these patients: The high morbidity and case fatality inherent to aneurysmal subarachnoid hemorrhage (SAH) must be carefully weighed against the relatively low rupture rate of UIAs and the potential morbidity associated with treatment—be it microsurgical or endovascular.^2-9^

Reliable scoring systems for risk of aneurysm growth and rupture have been developed in the Earlier subarachnoid hemorrhage, Location of aneurysm, Age, Population, Size of aneurysm, Shape of aneurysm score (ELAPSS) and Population, Hypertension, Age, Size of aneurysm, Earlier subarachnoid hemorrhage, Site of aneurysm score (PHASES), relying among others on proven risk factors such as posterior circulation location, larger diameter, higher age, and specific population and lifestyle factors.^2,5,10^ Still, objective assessment of treatment risks is necessary to make an educated recommendation toward treatment vs conservative management and toward microsurgical vs endovascular treatment. Various approaches toward stratifying patients into risk groups have been described and validated: The Unruptured Intracranial Aneurysm Treatment Score (UIATS) represents a formalized expert recommendation scoring system that provides guidance concerning conservative vs treatment strategies based on a consensus study.^3^ The SAFETEA^11^ risk score is based on 7 risk factors and reliably predicts the risk of death from any cause or clinical deterioration from neurological complications ≤30 days after microsurgical or endovascular treatment of UIAs but has not yet undergone external validation.

While such approaches are powerful tools to enable generalized and objective risk-benefit estimation, different patients harbor vastly different aneurysms and different patient characteristics which all influence treatment risks. Depending on aneurysm location, treatment risks are also vastly different for microsurgical vs endovascular treatment. Thus, in the era of “personalized/precision medicine”, it is imperative that we develop aides for accurate risk stratification in each individual patient, and for each treatment modality. Clinical prediction models enable generation of personalized risk estimates for each individual patient based on specific patient and aneurysm characteristics. While pilot studies have shown promising results in the prediction of UIA treatment risks,^12,13^ the most crucial aspect in the development of clinical prediction models is that they must be derived from and then externally validated in a multitude of different clinical settings, to rigorously test their real-world predictive performance.

## METHODS

Data were collected in an international consortium, the prediction of adverse events after microsurgery for intracranial unruptured aneurysms (PRAEMIUM) study (ClinicalTrials.gov: NCT04819074), to build a representative sample of patients who underwent microsurgical treatment of UIAs. This study adheres to Transparent Reporting of Imultivariable Prediction Outcome Diagnostics + Artificial Intelligence.^14^ Approval was obtained from local institutional review boards, and patients provided informed consent/informed consent was waived, depending on the local institutional review board.

### Inclusion/Exclusion

Patients operated microsurgically for UIAs from January 1, 2010, onward were considered eligible for inclusion to accurately represent the contemporary treatment paradigm. Only patients undergoing microsurgical treatment for 1 or multiple UIAs were included. Patients undergoing endovascular treatment were excluded, except those who underwent microsurgery, for example, a previously coiled aneurysm. Patients with previous SAH but undergoing microsurgical treatment of an unruptured, different aneurysm were included if treatment of the UIA occurred at least 4 weeks after SAH. Only expert microsurgical centers with adequate caseload (at least 100 UIAs treated microsurgically since January 1, 2010) were included.

### Data

Data were collected from prospective patient registries, from prospective registries supplemented by retrospectively collected variables, or retrospectively. All features were measured preoperatively. Recorded baseline variables included age, sex, maximum aneurysm diameter, anatomic location (artery), total number of aneurysms per patient, whether multiple aneurysms were treated during the index session, calcification of the aneurysm wall or neck, aneurysm morphology (saccular, dissecting, fusiform, or other such as blister or mycotic aneurysms), involvement of critical perforating or branch vessels, and intraluminal thrombosis. We also captured previous SAH, modified Rankin scale (mRS) at admission, previous aneurysm treatment, presence of anticoagulation/antiplatelet therapy preoperatively, and arterial hypertension, as well as American Society of Anesthesiologists grading, the PHASES,^5^ ELAPSS,^2^ and UIATS^3^ scores (including the UIATS subscores “favoring treatment” and “favoring conservative management”). Also included was the surgical approach: minimally invasive or standard approach and whether a bypass was performed.

### End Points

Three end points were defined to capture surgical risk holistically:

1. Neurological outcome, with favorable neurological outcome defined as a mRS of 0, 1, or 2.^15^
2. New sensorimotor neurological deficits.
3. All-cause adverse events, defined by the modified 2009 Clavien-Dindo grading (CDG).^16,17^ We defined any event with CDG of 1 or greater as adverse event. Surgery-related as well as non–surgery-related complications are counted. In case of multiple complications, only the complication with the highest CDG was counted per patient.

End points were assessed at discharge. For multiple aneurysms treated in 1 session, only 1 set of end points was recorded, based on the most complex treated aneurysm. If treated in separate sessions, end points were assessed at each discharge.

### Analysis

All analyses were performed in R 4.3.1.^18^ Numerical input variables were standardized using centering and scaling. Missing input data within the training sample assumed to be missing at random were imputed using a cotrained *k*-nearest neighbor algorithm.^19^ Gradient-boosting decision trees were applied.^20^ During training,^21^ hyperparameters were tuned using 5-fold cross-validation with 10 repeats, maximizing area under the curve (AUC). The threshold for binary classification was selected based on the “closest-to-(0,1)-criterion” and rounded.^22^ The models were then integrated into a web-app using the Shiny environment^23^ and underwent external validation. Quantile-based 95% CIs were obtained from 1000 bootstrap resamples. AUC-based variable importance is provided (**Supplementary Digital Content 1**, **Supplementary Table**, http://links.lww.com/NEU/F124).

## RESULTS

### Patient Cohort

Data on 3705 microsurgical procedures were collected from a total of 20 centers. Thirteen centers (2881, 78%) were used for model development. Fully trained models were evaluated on 824 patients (22%) from 7 additional centers. Patients were 56 ± 12 years old, and 1049 (28%) were male. Detailed demographic, aneurysmal, and surgical variables are provided (Table 1). At discharge, poor neurological outcome was seen in 514 patients (14%). New sensorimotor deficits were observed in 534 patients (14%), and 894 patients (24%) experienced any adverse events up until hospital discharge.

**TABLE 1.** Summary of Patient and Aneurysm Characteristics as Well as Outcome Measures at Discharge

Parameter	Overall	Development	External validation
No. of patients, n (%)	3705 (100)	2881 (77.8)	824 (22.2)
Age (y), mean (SD)	56.21 (11.56)	56.14 (11.69)	56.48 (11.09)
Male sex, n (%)	1049 (28.3)	817 (28.4)	232 (28.2)
mRS at admission, n (%)			
0	2161 (58.3)	1705 (59.2)	456 (55.3)
1	977 (26.4)	745 (25.9)	232 (28.2)
2	361 (9.7)	263 (9.1)	98 (11.9)
3	144 (3.9)	116 (4.0)	28 (3.4)
4	41 (1.1)	32 (1.1)	9 (1.1)
5	10 (0.3)	9 (0.3)	1 (0.1)
Missing	11 (0.3)	11 (0.4)	0 (0.0)
mRS at discharge, n (%)			
0	1850 (49.9)	1429 (49.6)	421 (51.1)
1	855 (23.1)	651 (22.6)	204 (24.8)
2	480 (13.0)	377 (13.1)	103 (12.5)
3	357 (9.6)	315 (10.9)	42 (5.1)
4	118 (3.2)	83 (2.9)	35 (4.2)
5	29 (0.8)	14 (0.5)	15 (1.8)
6	10 (0.3)	6 (0.2)	4 (0.5)
Missing	6 (0.2)	6 (0.2)	0 (0.0)
Arterial hypertension, n (%)	2071 (55.9)	1596 (55.4)	475 (57.6)
Anticoagulation/antiplatelet therapy, n (%)	887 (23.9)	762 (26.4)	125 (15.2)
Missing	1 (0.0)	1 (0.0)	0 (0.0)
American Society of Anesthesiologists score, n (%)			
1	646 (17.4)	431 (15.0)	215 (26.1)
2	1531 (41.3)	1140 (39.6)	391 (47.5)
3	1190 (32.1)	985 (34.2)	205 (24.9)
4	148 (4.0)	137 (4.8)	11 (1.3)
5	1 (0.0)	1 (0.0)	0 (0.0)
Missing	189 (5.1)	187 (6.5)	2 (0.2)
Prior subarachnoid hemorrhage, n (%)	474 (12.8)	368 (12.8)	106 (12.9)
Total number of aneurysms, mean (SD)	1.68 (1.11)	1.68 (1.11)	1.65 (1.10)
Multiple aneurysms treated during session, n (%)	738 (19.9)	616 (21.4)	122 (14.8)
Missing	3 (0.1)	3 (0.1)	0 (0.0)
Maximum aneurysm diameter (mm), mean (SD)	7.05 (4.88)	7.01 (4.86)	7.20 (4.96)
Anatomic location, n (%)			
Paraophthalmic ICA	316 (8.5)	294 (10.2)	22 (2.7)
ICA: PCom	261 (7.0)	191 (6.6)	70 (8.5)
ICA: other	323 (8.7)	258 (9.0)	65 (7.9)
ACA: proximal and ACom	673 (18.2)	505 (17.5)	168 (20.4)
ACA: distal	151 (4.1)	120 (4.2)	31 (3.8)
MCA: M1	130 (3.5)	101 (3.5)	29 (3.5)
MCA: bifurcation and distal	1903 (51.4)	1403 (48.7)	500 (60.7)
Posterior circulation	115 (3.1)	109 (3.8)	6 (0.7)
Other location	53 (1.4)	36 (1.2)	17 (2.1)
Calcification of wall or neck, n (%)	410 (11.1)	297 (10.3)	113 (13.7)
Missing	235 (6.3)	234 (8.1)	1 (0.1)
Aneurysm morphology, n (%)			
Saccular	3506 (94.6)	2719 (94.4)	787 (95.5)
Dissecting	15 (0.4)	11 (0.4)	4 (0.5)
Fusiform	127 (3.4)	100 (3.5)	27 (3.3)
Other	54 (1.5)	49 (1.7)	5 (0.6)
Missing	3 (0.1)	2 (0.1)	1 (0.1)
Involvement of critical perforating or branch vessels, n (%)	1240 (33.5)	895 (31.1)	345 (41.9)
Missing	11 (0.3)	10 (0.3)	1 (0.1)
Intraluminal thrombosis, n (%)	331 (8.9)	258 (9.0)	73 (8.9)
Missing	181 (4.9)	180 (6.2)	1 (0.1)
Prior aneurysm treatment, n (%)	319 (8.6)	260 (9.0)	59 (7.2)
Missing	1 (0.0)	1 (0.0)	0 (0.0)
Bypass necessary, n (%)	88 (2.4)	69 (2.4)	19 (2.3)
Missing	1 (0.0)	1 (0.0)	0 (0.0)
PHASES score, mean (SD)	4.95 (3.03)	4.88 (3.00)	5.23 (3.13)
ELAPSS score, mean (SD)	15.55 (7.68)	15.25 (7.64)	16.60 (7.73)
UIATS score for treatment, mean (SD)	11.67 (4.53)	11.83 (4.57)	11.13 (4.37)
UIATS score for conservative management, mean (SD)	9.71 (2.79)	9.64 (2.71)	9.95 (3.05)
New sensorimotor neurological deficit, n (%)	534 (14.4)	416 (14.4)	118 (14.3)
Missing	1 (0.0)	1 (0.0)	0 (0.0)
Poor neurological outcome (mRS ≥3), n (%)	514 (13.9)	418 (14.5)	96 (11.7)
Missing	6 (0.2)	6 (0.2)	0 (0.0)
All-cause adverse events (CDG ≥1), n (%)	894 (24.1)	646 (22.4)	248 (30.1)
Missing	1 (0.0)	1 (0.0)	0 (0.0)

ACA, anterior cerebral artery; ACom, anterior communicating artery; CDG, Clavien-Dindo grading; ELAPSS, Earlier subarachnoid hemorrhage, Location of aneurysm, Age, Population, Size of aneurysm, Shape of aneurysm score; ICA, internal carotid artery; MCA, middle cerebral artery; mRS, modified Rankin Scale; PCom, posterior communicating artery; PHASES, Population, Hypertension, Age, Size of aneurysm, Earlier subarachnoid hemorrhage, Site of aneurysm score; UIATS, Unruptured Intracranial Aneurysm Treatment Score.

### Performance Evaluation

#### Neurological Outcome

A predicted probability of >15% was considered high risk for poor neurological outcome. Based on this threshold, prediction of poor neurological outcome (mRS ≥3) at discharge achieved an external validation AUC of 0.70 (95% CI: 0.63-0.75) with sensitivity of 0.43 (95% CI: 0.33-0.53) and specificity of 0.83 (95% CI: 0.80-0.86) (Table 2). Regarding calibration (Figure), the model performed relatively well at external validation with an intercept of 0.18 (95% CI: −0.05 to 0.41) and slope of 0.73 (95% CI: 0.52-0.94).

**TABLE 2.** Discrimination and Calibration Metrics of the Machine Learning–Based Prediction Models for Outcomes at Discharge From Microsurgical Treatment of UIAs

Metric	Clinical prediction model					
	Poor neurological outcome (mRS ≥3)		New sensorimotor neurological deficit		Any adverse event (CDG ≥1)	
	Development	External validation	Development	External validation	Development	External validation
Model	Gradient boosted trees		Gradient boosted trees		Gradient boosted trees	
Dichotomization cutoff	0.15		0.15		0.20	
No. observations	2875	824	2880	824	2880	824
Discrimination						
AUC	0.77 (0.76-0.78)	0.70 (0.63-0.75)	0.68 (0.67-0.69)	0.69 (0.63-0.74)	0.58 (0.57-0.59)	0.59 (0.55-0.64)
Accuracy	0.72 (0.72-0.73)	0.78 (0.76-0.81)	0.69 (0.68-0.69)	0.76 (0.73-0.78)	0.53 (0.52-0.53)	0.55 (0.52-0.58)
Sensitivity	0.70 (0.69-0.72)	0.43 (0.33-0.53)	0.56 (0.55-0.58)	0.41 (0.33-0.51)	0.61 (0.60-0.62)	0.57 (0.51-0.63)
Specificity	0.73 (0.72-0.73)	0.83 (0.80-0.86)	0.71 (0.70-0.71)	0.81 (0.78-0.84)	0.50 (0.50-0.51)	0.54 (0.50-0.58)
PPV	0.30 (0.30-0.31)	0.25 (0.18-0.31)	0.25 (0.24-0.26)	0.27 (0.21-0.34)	0.26 (0.26-0.27)	0.35 (0.30-0.39)
NPV	0.93 (0.93-0.94)	0.92 (0.89-0.94)	0.91 (0.90-0.91)	0.89 (0.87-0.91)	0.82 (0.81-0.82)	0.74 (0.70-0.78)
F1 score	0.82 (0.81-0.82)	0.87 (0.85-0.89)	0.80 (0.79-0.80)	0.85 (0.83-0.87)	0.62 (0.62-0.63)	0.62 (0.59-0.66)
Calibration						
Intercept	0.01 (−0.02 to 0.05)	0.18 (−0.05 to 0.41)	0.01 (−0.02 to 0.05)	0.24 (0.04-0.44)	0.01 (−0.02 to 0.04)	0.47 (0.31-0.62)
Slope	0.96 (0.92-0.99)	0.73 (0.52-0.94)	0.85 (0.81-0.89)	0.94 (0.66-1.22)	0.57 (0.52-0.62)	0.75 (0.44-1.05)

AUC, area under the curve; CDG, Clavien-Dindo grading; mRS, modified Rankin Scale; NPV, negative predictive value; PPV, positive predictive value; UIA, unruptured intracranial aneurysm.

Metrics are provided with bootstrapped 95% CIs based on 1000 samples with replacement. Reported development performance is the resampled cross-validation performance.

**FIGURE.**
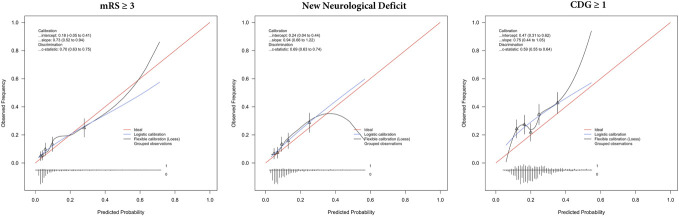
Calibration curves of the 3 clinical prediction models for poor neurological outcome at discharge (mRS ≥3), new sensorimotor neurological deficits at discharge, and occurrence of any adverse event (CDG ≥1) up to discharge. Performance when testing the fully trained models on the external validation cohort of 824 patients is reported. The predicted probabilities are distributed into 5 equally sized groups and contrasted with the actually observed frequencies of the binary outcomes. Calibration intercept and slope are calculated. A perfectly calibrated model has a calibration intercept of 0 and slope of 1. Metrics are provided with bootstrapped 95% CIs. CDG, Clavien-Dindo grading; mRS, modified Rankin Scale.

#### New Neurological Deficits

A predicted probability of >15% was considered high risk for new sensorimotor neurological deficits. Based on this threshold, prediction of new sensorimotor neurological deficits at discharge achieved an external validation AUC of 0.69 (95% CI: 0.63-0.74) with sensitivity of 0.41 (95% CI: 0.33-0.51) and specificity of 0.81 (95% CI: 0.78-0.84). Regarding calibration, the model performed relatively well at external validation with an intercept of 0.24 (95% CI: 0.04-0.44) and slope of 0.94 (95% CI: 0.66-1.22).

#### All-Cause Adverse Events

A predicted probability of >20% was considered high risk for all-cause adverse events. Based on this threshold, prediction of all-cause adverse events (CDG ≥1) at discharge achieved an external validation AUC of 0.59 (95% CI: 0.55-0.64) with sensitivity of 0.57 (95% CI: 0.51-0.63) and specificity of 0.54 (95% CI: 0.50-0.58). The model exhibited fair calibration at external validation with an intercept of 0.47 (95% CI: 0.31-0.62) and slope of 0.75 (95% CI: 0.44-1.05).

### Deployment

The prediction model is accessible at https://neurosurgery.shinyapps.io/PRAEMIUM/.

## DISCUSSION

As the number of incidentally detected UIAs is steadily increasing,^24^ the discussion on whether to treat and then how to treat has become a central issue for specialists and patients. Concerning treatment vs observation, no level I evidence from controlled trials exists. Guidelines recommend preventive treatment if rupture risk outweighs treatment risks. An increased rupture risk is considered particularly in patients with larger aneurysms, documented aneurysm enlargement, previous SAH, or familial aneurysms.^24,25^ Accurate prediction of the risk of rupture are thus key components in managing patients with UIAs. The PHASES^5^ score is an established risk stratification for rupture risk, as is the ELAPSS^2^ score for risk of aneurysm growth. The correct and unbiased estimation of treatment risk is equally important in the delicate equation of treatment decisions for patients with UIAs. The UIATS^3^ is an expert consensus-based score that combines expected rupture and treatment risks into a holistic recommendation toward observation vs treatment. These efforts, taken together, have established a toolkit for patient counseling and risk-benefit discussion in neurovascular interdisciplinary boards.

A fully personalized and precise prediction for each individual patient—objectively forecasting risks of treatment, including neurological deterioration and adverse events—requires new tools in our armamentarium. These tools must be specific and analyze microsurgical treatment separately from endovascular treatment. In recent years, the role of microsurgery in the treatment of UIAs has shifted toward anterior circulation aneurysms and toward younger patients with good clinical status^24,25^—this occurred after the wide introduction of endovascular coil embolization for ruptured aneurysms after the International Subarachnoid Aneurysm Trial in 2002. Endovascular techniques have since then also been applied to UIAs.^26^ For UIAs, there are only few high-quality data from controlled studies comparing endovascular vs surgical treatment. A recent pragmatic randomized trial by Darsaut et al^27^ compared clipping vs endovascular treatment of UIAs and found that a composite primary outcome defined as failure of aneurysm occlusion, intracranial hemorrhage during follow-up, or residual aneurysms at 1 year was significantly lower in microsurgically treated patients, particularly those with middle cerebral artery (MCA) aneurysms. Still, we owe the patient a more personalized and precise prediction of the treatment risk, taking into account the complexity of the angioanatomy, of the proposed treatment strategy, the patient's own risk factors, and the treating center's own experience and expertise.^24,25^ This is particularly true for patients with UIAs, since these patients usually walk in with no symptoms (in case of incidental aneurysms) or with slight focal but usually reversible symptoms (in case of symptomatic aneurysms with mass effect).

In patients with UIAs, the main goal of preventive treatment must thus be to “do no harm”, and this is precisely where clinical prediction modeling is most effective. Predicting surgical outcomes is a difficult task, as a multitude of measurable and nonmeasurable factors influence outcomes and adverse events. Approaches based on machine learning proved beneficial in generating accurate predictions, but must be rigorously externally validated before deployment, and should be both reproducible and explainable.^28^ Because—fortunately—adverse outcomes after treatment of UIAs are relatively rare, large sample sizes are necessary. In addition, surgical complexity between a small saccular MCA aneurysm and a paraophthalmic or giant basilar tip aneurysm is radically different. Surgical expertise strongly influences outcomes and remains difficult to capture objectively, especially from the patient's perspective.^29^ These factors make development of clinical prediction models that generalize well to an external validation cohort both essential and challenging. Previous efforts have included 2 pilot studies with smaller numbers, one of which with external validation, which was unsuccessful.^12,13^ The SAFETEA^11^ score is a powerful tool that achieved a training AUC of 0.72 for both microsurgical and endovascular treatment for predicting death from any cause or clinical deterioration from neurological complications ≤30 days,. However, the SAFETEA score has not yet undergone external validation and thus cannot be applied in clinical practice.

The PRAEMIUM study represents an international effort toward developing evidence-based decision-support tools in the microsurgical treatment of UIAs. Moreover, the PRAEMIUM data set represents the largest collection of outcome data of patients with UIAs undergoing microsurgical treatment that exists in the literature. Benchmarking surgical procedures is important and allows for intercenter comparisons. A recent study by Drexler et al^9^ found that benchmark outcomes should include a favorable neurological outcome (mRS ≤2) in over 95% of patients, an adverse events rate of under 21%, and new motor neurodeficits under 6%. A meta-analysis by Algra et al^30^ estimated the pooled adverse event rate for microsurgery of UIAs at 8.34%. The end points described within our study compare favorably with these literature estimates and indicate that the PRAEMIUM cohort could serve as another benchmark for the microsurgical treatment of UIAs.

After extensive external validation, the 2 models for poor neurological outcome and for new sensorimotor neurological deficits demonstrated relatively good calibration—meaning that their predicted probabilities correspond well to the actually observed rates of poor outcomes. This is also visible within the calibration plots. However, binary discrimination performance—as measured by AUC, specificity, negative predictive value—in ruling out poor outcomes was only fair. Well-calibrated models are clinically valuable (as opposed to models with high discrimination but poor calibration).^31^ This is likely because, ultimately, prediction of surgical complications in individual patients is likely impossible with perfect accuracy. Instead, each patient has a spectrum of risk based on their demographics, anatomic-pathological characteristics, and the treatment options. Because the PRAEMIUM models for poor neurological outcome and new deficits at discharge seem to be well-calibrated, we suggest that these models could be introduced into clinical practice—not as a replacement of expert judgment but as an adjunct, as a kind of “digital objective second opinion” for the risks of microsurgical treatment. The predicted probabilities generated by these models could be useful in weighing risks and benefits of microsurgical treatment vs observation, especially in combination with established scores for rupture and growth risk.^2,5^ However, binary predictions generated by such models should be interpreted much more cautiously than the predicted probabilities themselves. For example, a binary prediction suggesting that a specific patient may experience a poor neurological outcome should be interpreted as the patient being at significantly increased risk (ie, in this study a risk of >15%) but not as an absolute “yes or no” prediction, which would require much higher discrimination performance.

The model was unable to predict the occurrence of all-cause adverse events with high certainty: Discrimination performance here was poor, although the predicted probabilities still seem to somewhat correlate with real-world risk for adverse events, indicating fair calibration. Consequently, the model for adverse events is less likely to be useful in clinical practice. The most likely reason for the markedly poorer performance is that the definition of adverse events according to the established CDG^16,17^ includes a wide variety of different events such as rebleeding, pulmonary thromboembolism, or wound infections. These events may be influenced by various, sometimes opposing, risk factors, making their accurate prediction a formidable challenge.

### Limitations

Our data represent a mix of prospectively and retrospectively collected patient data. In addition, data were collected only from European, North American, and Australian expert microsurgical centers with a regular caseload of UIA clipping. Information on intraoperative techniques such as electrophysiological monitoring and fluorescence video-angiography was not included.

Current clinical prediction models are still limited by manual data collection and expert classifications, for example, what is calcified and what is not considered calcified, and manual measurements, for example, aneurysm size. Such measurements do not exhibit perfect inter-rater agreement^32^ and thus may be collected differently by various raters in various centers. Similarly, while feature and end point definitions were standardized across all centers, treatment protocols were not. Because case complexity and outcomes do differ among centers,^9^ our prediction models are unable to take into account each individual center's or surgeon's expected outcome. Furthermore, calibration of prediction models is highly dependent on the incidence of end points within different cohorts.^33^ In the end, all of these inconsistencies are the reason why rigorous external validation is crucial: By testing the fully developed models on new data from 7 different centers, we can better assess the expected real-world performance of our prediction tool.

A minority of included patients harbored posterior circulation aneurysms, highly complex aneurysms, or underwent trapping with bypass. Although our models do take into account these differences, they are inherently biased toward the most seen aneurysms such as those of the MCA or ACom. Last, our end points are collected at discharge. Although neurological outcome and deficits often improve over time, we purposefully chose to collect discharge status as patients with UIAs are usually asymptomatic before treatment and should remain so afterward.

## CONCLUSIONS

The developed models for prediction of poor neurological outcome and new sensorimotor neurological deficits at discharge exhibit good calibration and fair discrimination based on an international external validation, indicating that the predicted probabilities correspond well to real-world risks and may thus be clinically useful in more objectively estimating the risk of microsurgical treatment. We were not able to predict all-cause adverse events with similarly high performance. Preoperative prediction of treatment risk remains a formidable challenge, often relying not only on evidence but also on experience and expertise. The PRAEMIUM tool offers an attempt to make the art of prediction and patient counseling more objective and personalized, benefitting both patients and specialists.

## Supplementary Material

## SUPPLEMENTAL DIGITAL CONTENT

**Supplementary Digital Content 1: Supplementary Table.** Variable importance measures of the 3 fully trained clinical prediction models. The reported values are AUC-based and rounded to 2 decimals. Variables with very rare occurrence were not considered by the model. ACA, anterior cerebral artery; ACom, anterior communicating artery; ASA, American Society of Anesthesiologists Score; CDG, Clavien-Dindo grading; ELAPSS, Earlier subarachnoid hemorrhage, Location of aneurysm, Age, Population, Size of aneurysm, Shape of aneurysm score; ICA, internal carotid artery; MCA, middle cerebral artery; mRS, modified Rankin Scale; PCom, posterior communicating artery; PHASES, Population, Hypertension, Age, Size of aneurysm, Earlier subarachnoid hemorrhage, Site of aneurysm score; UIATS, Unruptured Intracranial Aneurysm Treatment Score.
