# Supplementary material for: Risk Models for Adverse Events in Microsurgery for Intracranial Unruptured Aneurysms
Source: Neurosurgery. 2025 Dec 5;99(2):452–60. doi: 10.1227/neu.0000000000003867 (PMC13336704; doi:10.1227/neu.0000000000003867)
Supplement: Supplementary file 1 [file neu-99-452-s001.docx]

**Supplementary Table 1** Variable importance measures of the three fully trained clinical prediction models. The reported values are AUC-based and rounded to two decimals. Variables with very rare occurrence were not considered by the model.

|  | **Clinical Prediction Model** | | |
| --- | --- | --- | --- |
| **Parameter** | **Poor Neurological Outcome (mRS ≥ 3)** | **New Sensorimotor Neurological Deficit** | **Any Adverse Event (CDG ≥ 1)** |
| Age | 0.12 | 0.17 | 0.17 |
| Male sex | 0.01 | 0.03 | 0.02 |
| Modified Rankin Scale (mRS) at admission |  |  |  |
| 0 |  |  |  |
| 1 | 0.01 | 0.03 | 0.02 |
| 2 | 0.00 | 0.01 | 0.01 |
| 3 |  |  |  |
| 4 |  |  |  |
| 5 |  |  |  |
| Arterial hypertension | 0.01 | 0.01 | 0.01 |
| Anticoagulation / antiplatelet therapy | 0.00 | 0.01 | 0.02 |
| American Society of Anesthesiologists (ASA) Score |  |  |  |
| 1 | 0.09 | 0.02 | 0.00 |
| 2 | 0.12 | 0.04 | 0.02 |
| 3 | 0.12 | 0.06 | 0.02 |
| 4 |  |  |  |
| 5 |  |  |  |
| Prior subarachnoid hemorrhage | 0.02 | 0.00 | 0.01 |
| Total number of aneurysms | 0.03 | 0.06 | 0.04 |
| Multiple aneurysms treated during session | 0.03 | 0.04 | 0.01 |
| Maximum aneurysm diameter | 0.15 | 0.19 | 0.23 |
| Anatomical location |  |  |  |
| Paraophthalmic ICA | 0.01 | 0.01 | 0.00 |
| ICA: PCom | 0.00 | 0.00 | 0.00 |
| ICA: other | 0.00 | 0.00 | 0.00 |
| ACA: proximal and ACom | 0.00 | 0.00 | 0.00 |
| ACA: distal |  |  |  |
| MCA: M1 |  |  |  |
| MCA: bifurcation and distal | 0.02 | 0.03 | 0.01 |
| Posterior circulation |  |  |  |
| Other location |  |  |  |
| Calcification of wall or neck | 0.01 | 0.00 | 0.00 |
| Aneurysm morphology |  |  |  |
| Saccular | 0.01 | 0.01 | 0.00 |
| Dissecting |  |  |  |
| Fusiform |  |  |  |
| Other |  |  |  |
| Involvement of critical perforating or branch vessels | 0.01 | 0.01 | 0.02 |
| Intraluminal thrombosis | 0.00 | 0.01 | 0.02 |
| Prior aneurysm treatment | 0.01 | 0.03 | 0.01 |
| Bypass necessary |  |  |  |
| PHASES score | 0.02 | 0.03 | 0.10 |
| ELAPSS score | 0.08 | 0.08 | 0.05 |
| UIATS score for treatment | 0.08 | 0.08 | 0.10 |
| UIATS score for conservative management | 0.03 | 0.04 | 0.10 |

*mRS, modified Rankin Scale; CDG, Clavien-Dindo Grading; ASA, American Society of Anesthesiologists Score; ICA, internal carotid artery; PCom, posterior communicating artery; ACA, anterior cerebral artery; ACom, anterior communicating artery; MCA, middle cerebral artery;* *PHASES, Population, Hypertension, Age, Size of aneurysm, Earlier subarachnoid hemorrhage, Site of aneurysm score; ELAPSS, Earlier subarachnoid hemorrhage, Location of aneurysm, Age, Population, Size of aneurysm, Shape of aneurysm score; UIATS, Unruptured Intracranial Aneurysm Treatment Score.*
